# Perinatal Nicotine Exposure Increases Angiotensin II Receptor-Mediated Vascular Contractility in Adult Offspring

**DOI:** 10.1371/journal.pone.0108161

**Published:** 2014-09-29

**Authors:** DaLiao Xiao, Chiranjib Dasgupta, Yong Li, Xiaohui Huang, Lubo Zhang

**Affiliations:** Center for Perinatal Biology, Department of Basic Sciences, Loma Linda University School of Medicine, Loma Linda, California, United States of America; The University of Manchester, United Kingdom

## Abstract

Previous studies have reported that perinatal nicotine exposure causes development of hypertensive phenotype in adult offspring.

**Aims:**

The present study was to determine whether perinatal nicotine exposure causes an epigenetic programming of vascular Angiotensin II receptors (ATRs) and their-mediated signaling pathway leading to heightened vascular contraction in adult offspring.

**Main methods:**

Nicotine was administered to pregnant rats via subcutaneous osmotic minipumps from day 4 of gestation to day 10 after birth. The experiments were conducted at 5 months of age of male offspring.

**Key Findings:**

Nicotine treatment enhanced Angitension II (Ang II)-induced vasoconstriction and 20-kDa myosin light chain phosphorylation (MLC_20_-P) levels. In addition, the ratio of Ang II-induced tension/MLC-P was also significantly increased in nicotine-treated group compared with the saline group. Nicotine-mediated enhanced constrictions were not directly dependent on the changes of [Ca^2+^]_i_ concentrations but dependent on Ca^2+^ sensitivity. Perinatal nicotine treatment significantly enhanced vascular ATR type 1a (AT_1a_R) but not AT_1b_R mRNA levels in adult rat offspring, which was associated with selective decreases in DNA methylation at AT_1a_R promoter. Contrast to the effect on AT_1a_R, nicotine decreased the mRNA levels of vascular AT_2_R gene, which was associated with selective increases in DNA methylation at AT_2_R promoter.

**Significance:**

Our results indicated that perinatal nicotine exposure caused an epigenetic programming of vascular ATRs and their-mediated signaling pathways, and suggested that differential regulation of AT_1_R/AT_2_R gene expression through DNA methylation mechanism may be involved in nicotine-induced heightened vasoconstriction and development of hypertensive phenotype in adulthood.

## Introduction

Maternal cigarette smoking is the single most widespread perinatal insult in the world [1]. Epidemiological studies have shown that maternal smoking is associated with increased risk of cardiovascular disease in offspring later in life [1]–[3]. As one of the major active components in cigarette smoking, nicotine readily crosses the placenta and produces higher nicotine concentrations in the fetal circulation than that experienced by the mother [4]. The heightened nicotine concentration may contribute to the maternal cigarette smoking-induced developmental programming of cardiovascular dysfunction in offspring. Indeed, nicotine use during pregnancy can cause cardiovascular disorders and hypertension of offspring in different animal models [5]–[7]. Our recent studies have demonstrated that perinatal nicotine exposure reprograms cardiovascular functions and causes an exaggerated vascular reactivity and heightened blood pressure (BP) response in adult offspring [8]–[13]. However, the epigenetic and molecular mechanisms underlying the perinatal nicotine-induced heightened vascular reactivity in offspring are not fully understood.

It has been well documented that Angiotensin II (Ang II) has been implicated in pathophysiology of hypertension and other cardiovascular dysfunction induced by adverse *in utero* environment during the fetal development [14]–[16]. In an animal model of dietary restriction during pregnancy, elevated BP in adult offspring was associated with an increased sensitivity to Ang II [16]. Inhibition of Ang II type 1 receptor (AT_1_R) in early postnatal life following maternal dietary restriction prevented development of hypertension in adult offspring [16], [17]. In addition, a differential increase in the AT_1_R and decrease in AT_2_R expression has been reported at an early age of spontaneously hypertensive rats [18]. Our recent studies have demonstrated that perinatal nicotine exposure enhances AT_1_R but decreases AT_2_R protein levels in the vasculatures, resulting in a significant increased vascular reactivity and elevated BP response to Ang II in adult offspring [9]. Furthermore, previous studies have demonstrated that fetal nicotine exposure impairs kidney development and reprograms renal ATR expression which may contribute to fetal programming of hypertension [19], [20]. These studies suggest that the programming of Ang II receptor-mediated signaling pathways is a mechanistic link between programmed cardiovascular dysfunction and intrauterine adverse factors during early development. However, the mechanisms underlying perinatal nicotine-mediated Ang II receptors expressions and transduction signaling in the developing vasculatures remain elusive.

The present study was designed to test the hypothesis that maternal nicotine administration during pregnancy causes a differential epigenetic regulation of AT_1_R and AT_2_R gene expression via DNA methylation mechanism, leading to increase in down-stream signaling transduction pathway and enhance vascular reactivity in adult offspring. The specific aims of the present study were to determine in adult offspring whether and to what extent perinatal nicotine exposure affects 1) arterial mRNA levels of AT_1_R and AT_2_R, 2) DNA methylation levels in specific CpG sites at AT_1_R and AT_2_R promoter regions, 3) Ang II-induced intracellular Ca^2+^ ([Ca^2+^]_i_) signaling, 4) 20-kDa myosin light chain phosphorylation (MLC_20_-P) levels, and 5) vascular contractile function.

## Material and Methods

### Experimental animals

All procedures and protocols were approved by the Institutional Animal Care and Use Committee of Loma Linda University, and followed the guidelines by the National Institutes of Health Guide for the Care and Use of Laboratory Animals. Time-dated pregnant Sprague-Dawley rats were randomly divided into two groups: 1) saline control; and 2) nicotine administration through an osmotic minipump at 4 µg/kg/min from day 4 of pregnancy to day 10 after birth, as previously described [8]–[13]. In brief, on the fourth day of pregnancy, rats were lightly anesthetized with ketamine and xylazine, and an incision was made on their back to insert osmotic minipumps (Type 2ML4). The incision was closed with four sutures. Twelve pregnant rats were implanted with minipumps containing nicotine solution, and other eleven pregnant rats were implanted with minipumps containing only saline which served as the vehicle control. A total of 134 pups from the control and 141 pups from nicotine-treated pregnant rats were delivered. Our previous studies [8], [9], [12] and current studies did not show any significant differences in litter size following the nicotine exposure. Therefore, the litter size was intact as nature in each dam and all of the newborn pups were kept with their mothers until weaning. At weaning (3-weeks age), the male and female offspring were separated. Because our previous studies have demonstrated that fetal nicotine exposure causes a hypertensive response in male but not female offspring [9], the male offspring were kept and used for present studies at 5 months of age. Male offspring were anesthetized with ketamine and xylazine and scarified by removing the heart, and aortas or mesenteric arteries were isolated for functional and molecular biological studies at 5 months of age. In each experiment, the sample sizes represented the number of animal used and each animal was from a different litter.

### Contractions of aortic rings

At 5 months of age, aortas were isolated and cut into 4-mm rings and mounted in 10-ml tissue baths containing modified Krebs solution equilibrated with a mixture of 95%O_2_ and 5%CO_2._ Isometric tensions were measured at 37°C, as described previously [9]. After 60 min of equilibration, each ring was stretched to the optimal resting tension as determined by the tension developed in response to 120 mmol/L KCl added at each stretch level. Tissue were then stimulated with different doses of Angiotensin II (Ang II, 10^−9^∼10^−6^ M) (Sigma; St. Louis, MO), and contractile tensions and 20-kDa myosin light chain phosphorylation (MLC_20_-P) levels were measured simultaneously in the same tissues. Tensions developed were continuously recorded with an online computer. To measure phosphorylated MLC_20_ simultaneously in the same tissue, arterial rings were snap frozen with liquid N_2_-cooled clamps at the indicated times and were rapidly immersed in a dry ice-acetone slurry that contained a 10% trichloroacetic acid (TCA) and 10 mM DTT mixture. Tissues were then stored at −80°C until analysis of MLC_20_ phosphorylation.

### Measurement of MLC_20_ phosphorylation

Tissue MLC_20_ phosphorylation levels were measured as described previously [21]. Briefly, tissues were brought to room temperature in a dry ice-acetone-TCA-DTT mixture and then washed three times with ether to remove the TCA. Tissues were then extracted in 100 µL of sample buffer containing 20 mM Tris base and 23 mM glycine (pH 8.6), 8.0 M urea, 10 mM DTT, 10% glycerol and 0.04% bromophenol blue, as previously described [19]. Samples (20 µL) were electrophoresed at 12 mA for 2.5 h after a 30 min pre-run in 1.0 mm mini-polyacrylamide gels containing 10% arcelamide, 0.27% bisacrylamide, 40% glycerol and 20 mM Tris Base (pH 8.8). Proteins were transferred to nitrocellulose membranes and subjected to immunoblot with a specific MLC_20_ antibody (1:500, Sigma, St. Louis, MO). Goat anti-mouse IgG conjugated with horseradish peroxidase was used as a secondary antibody (1:2000, Calbiochem). Bands were detected with enhanced chemiluminsecence (ECL), visualized on films and analyzed with the Kodak ID image analysis software. Moles of phosphate per mole of MLC_20_ (fraction of MLC_20_ phosphorylated) were calculated by dividing the density of the phosphorylated band by the sum of the densities of the phosphorylated plus the unphosphorylated bands.

### Contractions of pressurized small mesenteric arteries

Also at 5 months of age, the mesenteric arcade was excised and small mesenteric arteries (∼200 µm in diameter) were dissected out under a dissecting microscope. The arterial segments were mounted and pressurized in an organ chamber (Living Systems, Burlington, VT), as previously described [9]. Vascular intracellular Ca^2+^ concentrations ([Ca^2+^]_i_) were measured in the same tissues loaded with the Ca^2+^ indicator Fura 2-AM, as previously described [9]. The vessels were pressurized to 45 mmHg that was considered the optimum pressure as shown in previous studies [9]. The pressurized arteries were stimulated with KCl (120 mmol/L) or single Ang II concentrations (10^−7^ M) until the maximal decrease in arterial diameter was obtained. Arterial diameter and Ca^2+^ signal were recorded using the SoftEdge Data Acquisition Subsystem (IonOptix, Milton, MA), as described previously [9].

### Real-Time Reverse Transcription–Polymerase Chain Reaction (RT-PCR)

RNA was extracted from aortic rings and abundance of AT_1a_R, AT_1b_R, and AT_2_R mRNA was determined by real-time reverse transcription–polymerase chain reaction using an Icycler Thermal cycler (Bio-Rad, Hercules, CA), as described previously [22], [23]. The primers used were: AT_1a_R, 5′-ggagaggattcgtggcttgag-3′ (forward) and 5′-ctttctgggagggttgtgtgat-3′ (reverse); AT_1b_R, 5′-atgtctccagtcccctctca-3′ (forward) and 5′-tgacctcccatctccttttg-3′ (reverse); and AT_2_R, 5′-caatctggctgtggctgactt-3′ (forward) and 5′-tgcacatcacaggtccaaaga-3′ (reverse). Real-time reverse transcription–polymerase chain reaction was performed in a final volume of 25 µL. Each polymerase chain reaction mixture consisted of 600 nmol/L of primers, 33 U of M-MLV reverse transcriptase (Promega, Madison, WI), and iQ SYBR Green Supermix (Bio-Rad) containing 0.625 U Taq polymerase, 400 µmol/L each of dATP, dCTP, dGTP, and dTTP, 100 mmol/L KCl, 16.6 mmol/L ammonium sulfate, 40 mmol/L Tris-HCl, 6 mmol/L MgSO_4_, SYBR Green I, 20 nmol/L fluorescing, and stabilizers. The following reverse transcription–polymerase chain reaction protocol was used: 42°C for 30 minutes, 95°C for 15 minutes followed by 40 cycles of 95°C for 20 seconds, 56°C for 1 minute, 72°C for 20 seconds. Glyceraldehyde-3-phosphate dehydrogenase was used as an internal reference and serial dilutions of the positive control was performed on each plate to create a standard curve. Polymerase chain reaction was performed in triplicate, and threshold cycle numbers were averaged.

### Quantitative Methylation-Specific Polymerase Chain Reaction (qMS-PCR)

CpG methylation at rat AT_1a_R and AT_2_R gene promoter was determined as previously described [22], [23]. Briefly, genomic DNA were isolated from aortic rings from control and nicotine-treated 5 month-old offspring using a GenElute Mammalian Genomic DNA mini-Prep kit (Sigma), denatured with 2 N NaOH at 42°C for 15 min, and treated with sodium bisulfite at 55°C for 16h, as previously described [13], [22], [23]. Bisulfite treatment of DNA converted cytosines to uracils. However, methylated cytosines at CpG were not converted. DNA was purified with a Wizard DNA clean up system (Promega). The bisulfite-modified DNA was used as a template for PCR. Specific primers were designed to amplify the target regions of interest with unmethylated CpG by detecting uracils and those with methylated CpG by detecting cytosines. GADPH was used as an internal reference gene. Real-time methylation-specific PCR was performed using iCycler real-time PCR system (BioRad). Data were presented as the percentage of methylation of the regions of interest (methylated CpG/[methylated CpG + ummethylated CpG] X 100%). The significant differences in the level of methylation of individual CpG between control and nicotine treated offspring were tested by Student's unpaired t test.

### Statistical analysis

Data were presented as the mean ± SEM. The differences were evaluated for statistical significance (P<0.05) by ANOVA or by *t* test, where appropriate.

## Results

### Effects of nicotine on Ang II-induced contraction and MLC_20_ phosphorylation

**Figure 1:**
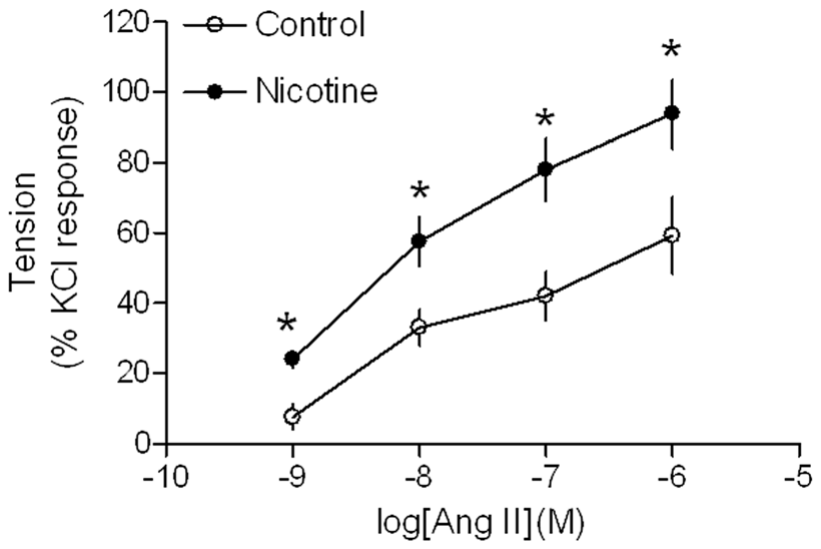
Effect of nicotine on Ang II-induced contractions in aortic rings in adult offspring. Ang II-induced contractions of aortic rings were determined in male adult offspring that had been exposed in utero to saline control or nicotine. Data are expressed as percentage of maximal KCl-induced contractions of the same tissue. Data are means ± SEM of tissues from saline control (n = 4) and nicotine (n = 5). The sample sizes represent the animal number from different litters of each group. Data were analyzed by student *t*-test at each correspondent Ang II dose point between saline control and nicotine-treated group. ^*^P <0.05 vs. control.

**Figure 2:**
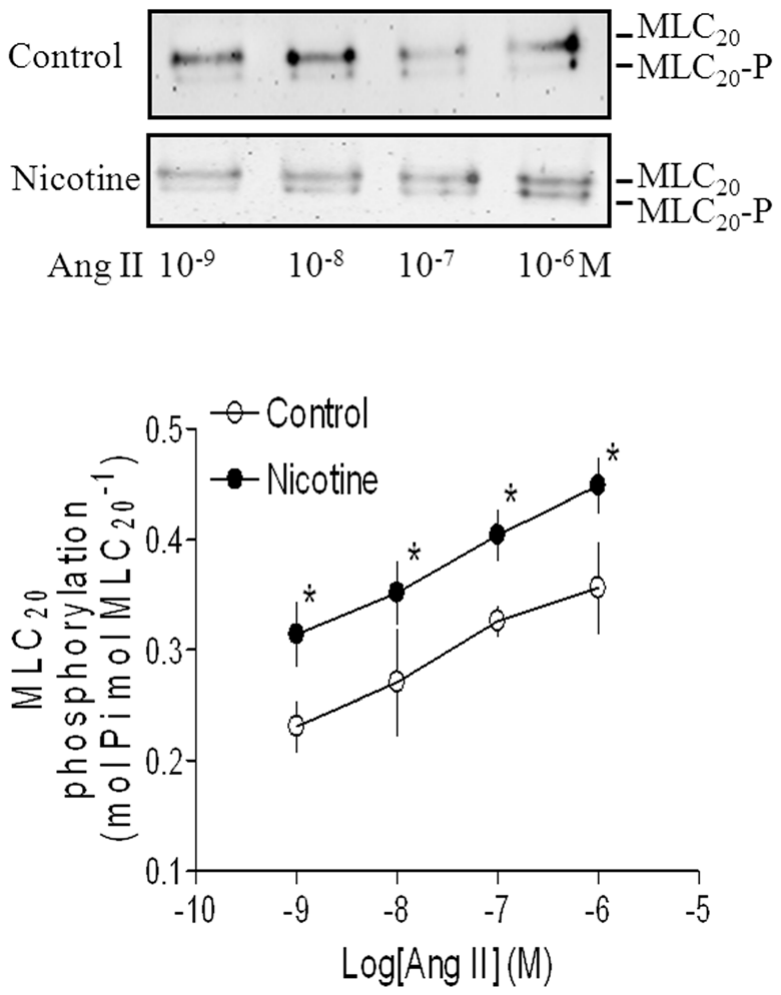
Effect of nicotine on Ang II-induced 20-kDa myosin light chain (MLC_20_) phosphorylation in aortic rings in adult offspring. Aortic rings from saline control and nicotine-treated groups were stimulated with increasing doses of Ang II. Phosphorylation of MLC_20_ (MLC_20_-P) was detected by Western blot (as described in METHODS). Representative immunoblot (top) shows unphosphorylated MLC_20_ and phosphorylated MLC_20_ (MLC_20_-P) induced by Ang II. Data are means ± SEM of tissues from saline control (n = 4) and nicotine (n = 5). The sample sizes represent the animal number from different litters of each group. Data were analyzed by student *t*-test at each correspondent Ang II dose point between saline control and nicotine-treated group. ^*^P <0.05 vs. control.

Ang II produced dose-dependent increases in contractions of aortic rings from both saline control and nicotine-treated animals (Fig. 1). However, Ang II-induced dose-dependent contractions were significantly higher in nicotine-treated group than those in saline control group. Similar to the contractions, Ang II-induced, dose-dependent increases in phosphorylation levels of MLC_20_ were also significantly enhanced in aortic rings of nicotine-treated rats compared with saline control rats (Fig. 2). To further determine whether nicotine affected the force-MLC_20_ phosphorylation relation, Ang II-induced tensions (Fig. 1) were plotted against their corresponding MLC_20_-P levels of both control and nicotine-treated groups (Fig. 2). The slope value obtained from the tension-MLC_20_-P relation curve in nicotine-treated group was significantly higher than that in control group (11.82 ± 1.1 vs. 7.04 ± 0.96, P <0.05).

### Effects of nicotine on Ang II-induced contraction and [Ca^2+^]_i_

**Figure 3:**
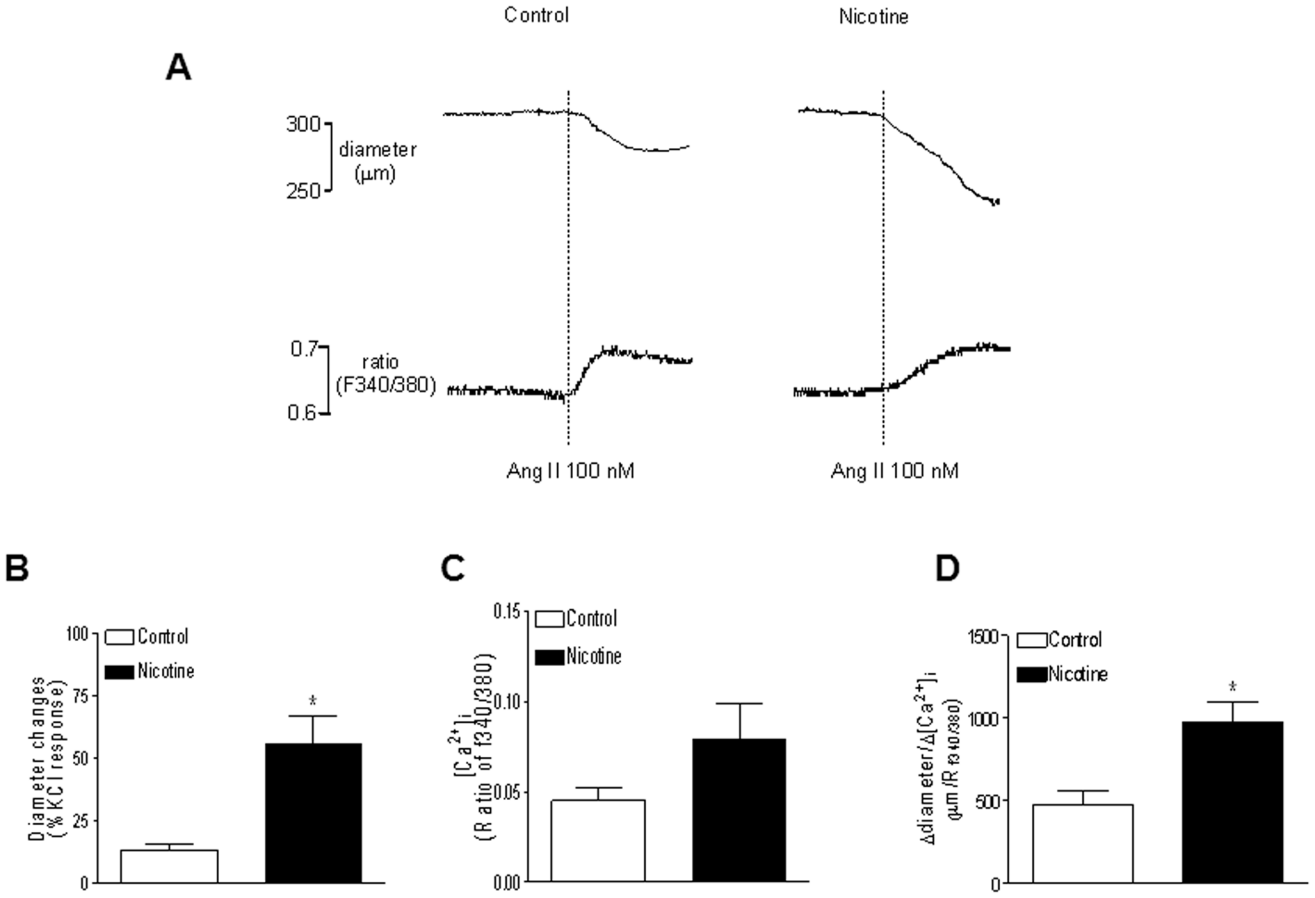
Effect of nicotine on Ang II-induced contractions and Ca^2+^ mobilization in mesenteric arteries in adult offspring. Ang II-induced contractions of pressurized mesenteric arteries were determined in male adult offspring that had been exposed in utero to saline control or nicotine. **A**, Typical recordings of Ang II (100 nM)-induced decreases in the arterial diameter and increments in [Ca^2+^]_i_ in the same tissue from saline control and nicotine-treated groups. Ang II-induced contractions (**B**), [Ca^2+^]_i_ (**C**), and arterial diameter change/[Ca^2+^]_i_ ratio (**D**) in mesenteric arteries from the control and nicotine-treated animals. Data are means ± SEM of tissues from saline control (n = 7) and nicotine (n = 8). The sample sizes represent the animal number from different litters of each group. Data were analyzed by student *t*-test. ^*^P <0.05 vs. control.

As shown in Figure 3A (trace), Ang II (100 nM) produced vasoconstrictions and decreases in the arterial diameter of pressurized small mesenteric arteries, which were associated with increases in [Ca^2+^]_i_. As shown in Figure 3 (bar graph), nicotine treatment significantly enhanced Ang II-induced contractile responses compared with the control group (Fig. 3B). Ang II-induced increases in [Ca^2+^]_i_ were not significant differences between nicotine-treated and control groups (Fig. 3C). However, the ratio of diameter change/[Ca^2+^]_i_-induced by Ang II was significantly higher in nicotine-treated group that that in control group (Fig. 3D) (974.4 ± 125.3 vs. 479.2 ± 81.8 µm/R_f340/f380_, P <0.05).

### Effect of nicotine on vascular expression patterns of AT_1_R and AT_2_R in adult offspring

**Figure 4:**
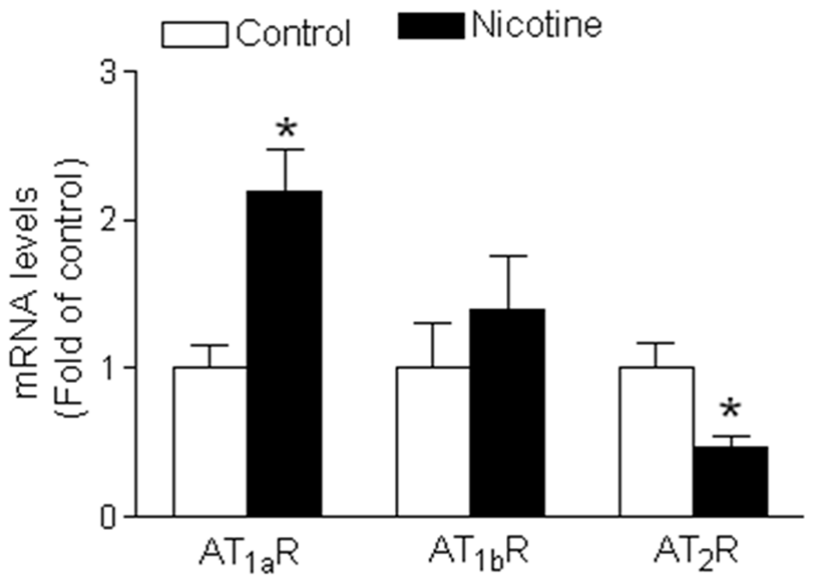
Effect of nicotine on mRNA abundance of AT_1_R and AT_2_R in aortic rings in adult offspring. RNA was extracted from aortic rings isolated from saline control and nicotine-treated animals. The abundance of AT_1a_R, AT_1b_R, and AT_2_R mRNA was determined by real-time reverse transcription-polymerase chain reaction analysis (as described in METHODS). Data are means ± SEM of tissues from saline control (n = 4) and nicotine (n = 4). The sample sizes represent the animal number from different litters of each group. Data were analyzed by student *t*-test. ^*^P <0.05 vs. control.

Our previous studies have demonstrated that perinatal nicotine exposure significantly increases AT_1_R but decreases AT_2_R protein levels, resulting in a significant increase in the ratio of AT_1_R/AT_2_R protein abundances in the aorta of adult male offspring [9]. To determine whether the protein expressions were regulated through transcriptional mechanism, we measured the mRNA abundance of ATR in the aorta of adult offspring. As shown in Figure 4, nicotine treatment significantly increased AT_1_aR mRNA but not AT_1b_R mRNA abundance in aorta as compared with the control. However, vascular AT_2_R mRNA abundance was significantly decreased in nicotine-treated group as compared with the control group.

### Effect of nicotine on DNA methylation of CpG locus at AT_1_R and AT_2_R promoter

**Figure 5:**
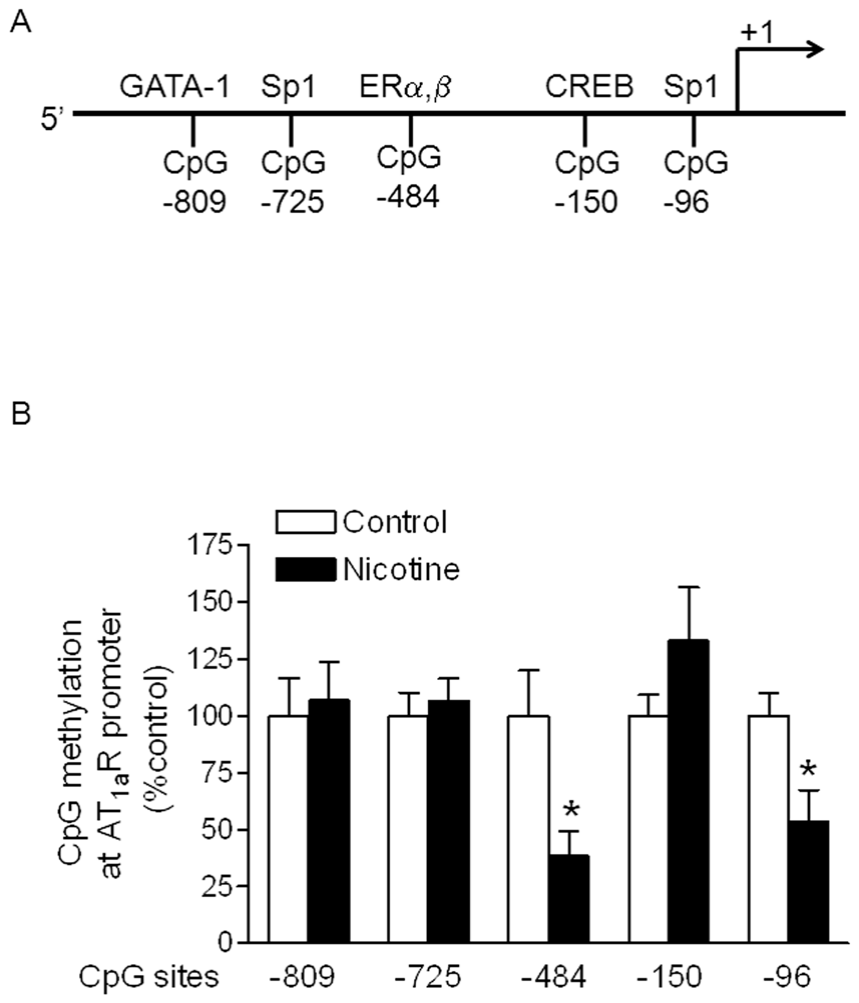
Effect of nicotine on CpG methylation of AT_1a_R promoter in aortic rings in male offspring. Aortic rings were freshly isolated from male adult offspring that had been exposed in utero to saline control or nicotine. DNA was isolated and methylation levels were determined by methylation-specific real-time PCR. Data are means ± SEM of tissues from saline control (n = 4) and nicotine (n = 4). The sample sizes represent the animal number from different litters of each group. Data were analyzed by student *t*-test. ^*^P <0.05 vs. control.

**Figure 6:**
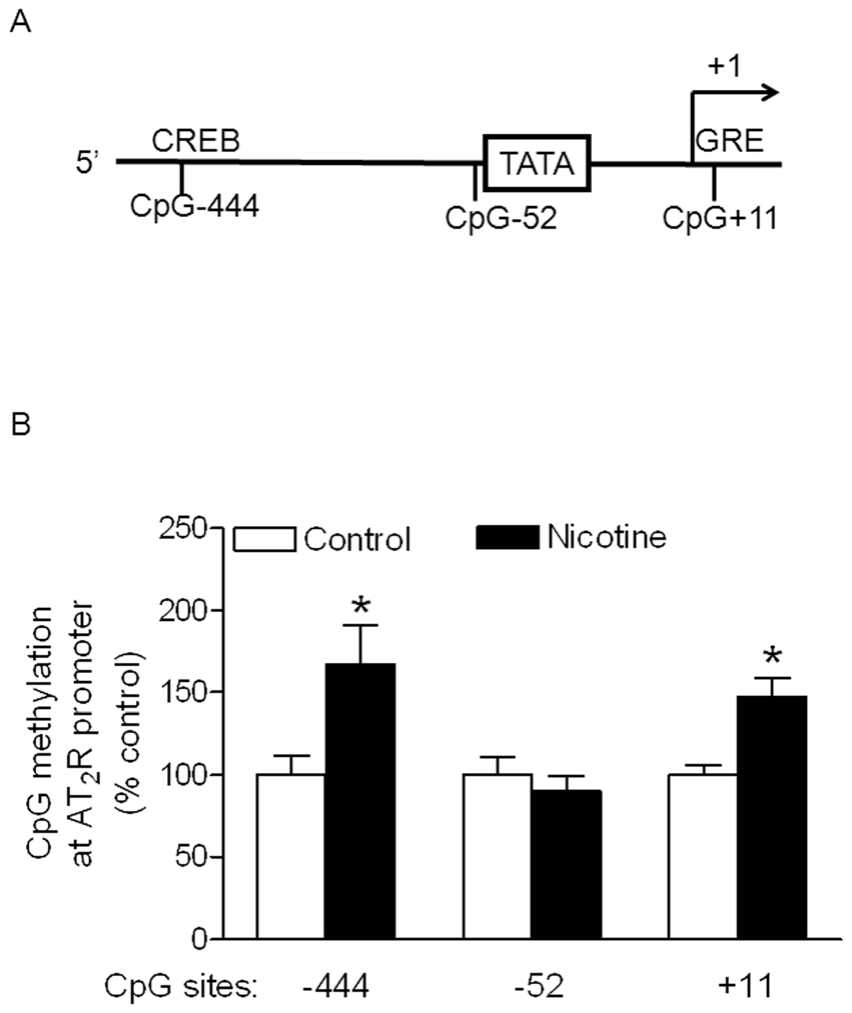
Effect of nicotine on CpG methylation of AT_2_R promoter in aortic rings in male offspring. Aortic rings were freshly isolated from male adult offspring that had been exposed in utero to saline control or nicotine. DNA was isolated and methylation levels were determined by methylation-specific real-time PCR. Data are means ± SEM of tissues from saline control (n = 4) and nicotine (n = 4). The sample sizes represent the animal number from different litters of each group. Data were analyzed by student *t*-test. ^*^P <0.05 vs. control.

Recent studies indicated that alteration of CpG methylation in sequence-specific transcription factor binding sites played an important role in epigenetic modification of gene expression patterns in the developing fetus in response to perinatal stress [13], [24]–[26]. From rat AT_1a_R gene bank, we have identified, at least five transcription factors binding sites located in CpG locus at rat AT_1a_R gene promoter region (Figure 5A). As shown in Figure 5B, the methylation levels of CpG locus at ERα, β binding site (−484) and Sp1 transcription factor binding site (−96) of AT_1a_R promoter region were significantly decreased in the aorta of adult offspring with maternal nicotine administration, when compared with the control. However, nicotine did not significantly alter the methylation status of CpG sites at −809, −725 and −150. In contrast to the effect of nicotine on AT_1a_R, nicotine selectively increased methylation levels of CpG locus at CREB binding site (−444) and GRE binding site (+11) of AT_2_R promoter region as compared with the control (Figure 6). However, the methylation levels at the CpG site (−52) near TATA box were not significant difference between the nicotine-treated and control animals.

## Discussion

Our previous studies have demonstrated that perinatal nicotine exposure causes a sex-dependent increase in blood pressure response in adult male rat offspring [9]. The present study provides new evidence that epigenetic programming of vascular Ang II receptors gene expressions and their-mediated signaling pathway plays a key role in nicotine-mediated developmental programming of hypertensive phenotype in adult offspring. The major findings in present study are the following: (1) perinatal nicotine exposure enhanced contractile responses to Ang II in both aortic and mesenteric arteries in adult offspring; (2) the enhanced vasoconstriction was not associated with changes of intracellular Ca^2+^ concentration ([Ca^2+^]_i_) but dependent on Ca^2+^ sensitivity of myofilaments; (3) nicotine enhanced Ang II-induced MLC_20_ phosphorylation level and the ratio of tension/MLC_20_-P; (4) nicotine exposure differentially increased AT_1a_R mRNA but decreased AT_2_R mRNA abundance in vasculatures; (5) the enhanced AT_1a_R gene expression was associated with decreased methylation levels of specific CpG sites at AT_1a_R promoter, and the decreased AT_2_R expression was associated with selectively increased methylation levels of specific CpG sites at AT_2_R promoter.

In the present study, we found that Ang II-induced vasoconstrictions were significantly increased in both aortic and mesenteric arteries of adult male offspring in perinatal nicotine treated group compared with the control group, which is consistent with previous findings that perinatal nicotine exposure enhances vasoconstriction and blood pressure response in male offspring in the similar animal model [8]–[10]. Heightened vascular contractility and programmed elevation of blood pressure has been reported in several different animal models, including in utero and neonatal exposure to secondhand smoke [27], antenatal glucocorticoid exposure [15] and maternal food restriction [28]. However, little is known about the programming of contractile signaling of vascular smooth muscle in response to in utero adverse environmental stress. Smooth muscle contraction is regulated through both thick and thin-filament regulatory pathways [29]–[31]. Thick-filament regulation is mediated through MLC_20_ phosphorylation-dependent pathway including both Ca^2+^-dependent and –independent mechanisms. Increased intracellular Ca^2+^ concentration leads to activation of myosin light chain kinase (MLCK) and phosphorylation of MLC_20_, but Ca^2+^-independent mechanisms mainly involve inactivation of myosin light chain phosphatase (MLCP) and decreased MLC_20_ dephosphorylation. Present findings that nicotine enhanced Ang II-induced vasoconstriction without significant change of intracellular Ca^2+^ concentration, suggesting that nicotine-mediated enhanced vasoconstriction may predominately regulated through Ca^2+^-independent mechanism, *i.e.* Ca^2+^ sensitivity of myofilaments. Because alterations in the activities of MLCK and/or MLCP at fixed [Ca^2+^]_i_ can alter the Ca^2+^ sensitivity of MCL_20_ phosphorylation, the present results that nicotine enhanced Ang II-induced MLC_20_ phosphorylation levels, suggest that nicotine exposure may regulate MLCK or/and MLCP activities independent of changes in [Ca^2+^]_i_. Previous studies have reported that MLCP is the primarily involved in agonist-induced Ca^2+^ sensitization, but MLCK is mainly regulated through agonist-induced changes in [Ca^2+^]_i_ [31]. These findings suggest that alteration of MLCP activity may play a key role in the regulation of the Ca^2+^ sensitivity of MLC_20_ phosphorylation in adaptation of vasculatures to nicotine exposure. Ang II-induced vascular contraction is regulated through G-protein coupled receptor-mediated signaling pathways [32]. Typically, Ang II binding with its receptor activates phospholipase C, leading to generate inositol trisphosphate (IP_3_) which increases in [Ca^2+^]_i_ and increase diacylglycerol (DAG) production which results in activation of protein kinas C (PKC). It has been demonstrated that PKC is able to modulate the Ca^2+^ sensitivity via phosphorylation of MLCP subunit, which leads to inhibition of MLCP activity [33]. Therefore, changes of Ang II/ATR-mediated PKC may play a key role in the adaptation of vascular thick-filament function to perinatal nicotine exposure.

In addition to the thick filament regulation, thin filament regulatory pathway also plays a key role in regulation of Ca^2+^ sensitivity. Smooth muscle thin filament-associated proteins, such as caldesmon, can inhibit myosin ATPase activity and generate vascular force without changes of [Ca^2+^]_i_ and MLC_20_ phosphorylation level [34]. In support this concept, our present results by experiments of simultaneous measurement of tension and MLC_20_ phosphorylation in the same tissue indicated that the ratio of Ang II-induced tension to MLC_20_ phosphorylation level was higher in nicotine-treated group than in saline control group, which suggest that thin filament regulatory pathway, *i.e.* increased tension at fixed MLC_20_-P, occurs in Ang II-mediated contraction and it is enhanced by nicotine exposure. In present study we can simultaneously measure the changes of pressurized arterial diameter and Ca^2+^ signal only in the small mesenteric artery (100∼200 µm in diameter) but not in the large aortic ring by using the IonOptix instrument system. However, we can simultaneously measure the changes of vascular tension and phosphorylation level of 20-kDa myosin light chain only in the aortic ring but not in the mesenteric artery in tissue bath. Therefore, we have to measure Ang II-induced changes of Ca^2+^ signal in mesenteric artery and but myosin light chain phosphorylation level in aortic ring. Although the technique limitation may affect our data interpretation, our previous studies have demonstrated that the effect of nicotine on Ang II-induced vascular contractile response is the same in both mesenteric artery and aortic ring [9]. This suggests that Ang II-induced signal response to nicotine exposure may be not changed by vessel type.

Our previous studies have demonstrated a significant increase in AT_1_R but decrease in AT_2_R protein abundances, resulting in a significant increase in the ratio of AT_1_R/AT_2_R of vasculatures in adult offspring in response to fetal nicotine exposure [9]. It suggests that epigenetic mechanisms are involved in reprogramming of the gene expression pattern of Ang II receptors in arteries that persist into adulthood. In the present studies, our data indicated that nicotine treatment significantly increased AT_1_aR mRNA but decreased AT_2_R mRNA abundance in vasculatures of adult offspring as compared with the control group, suggesting that nicotine-mediated alteration of ATR protein level is mainly regulated at transcriptional level. Change of transcriptional level of gene in fetal programming suggests epigenetic mechanisms involved. One of the major epigenetic mechanisms is DNA methylation. Recent studies have reported that DNA methylation is a key mechanistic link between cigarette smoking and cancer, as well as prenatal cigarette-smoke exposure and the development of adult chronic diseases [35]. DNA methylation is one of the most important mechanisms for epigenetic modification of gene expression patterns and occurs at cytosine in the CpG dinucleotide sequence [36], [37]. Methylation of CpG islands in gene promoter region can alter chromatin structure and transcription, leading to a long-term shutdown of the gene expression. In addition, methylation of a single CpG dinucleotide at sequence-specific transcription factor binding sites may inhibit gene expression through changes in the binding affinity of transcription factors [38]. Recent studies have demonstrated that CpG methylation in sequence-specific transcription factor binding sites play an important role in epigenetic modification of gene expression patterns in the developing fetus in response to different perinatal stresses [13], [24]–[26]. In the present study, we have identified several CpG islands located in the transcription factor binding sites in rat AT_1_aR promoter. Of these CpG sites, nicotine selectively decreased the methylation levels at -484 and -96 CpG locus, suggesting that hypomethylation of the selective CpG locus may be an important epigenetic mechanism in up-regulation of AT_1_R gene expression of vasculatures in response to perinatal nicotine exposure. Since CpG at the sites of -449 and -63 have been identified respectively as ERα,β and Sp1 transcription factor binding sites in rat AT_1_aR promoter, we might speculate that nicotine-mediated decrease in sequence-specific CpG methylation at ERα,β or Sp1 binding site may be a novel mechanism of altering those transcription factors binding affinity to AT_1_aR promoter and altering AT_1a_R promoter activity and gene expression. Indeed, previous studies have demonstrated that fetal stress-mediated increased sequence-specific CpG methylation at Sp1 and Egr1 binding sites at protein kinas Cε promoter results in decreased the transcription factors binding affinity and activity in PKCε promoter, leading to PKCε gene repression in the developing heart [13], [24], [25].

The effect of nicotine on ERα,β binding sites within AT_1_aR promoter is interesting and may have significant implication. Epidemiological evidence indicates that the incidence of cardiovascular disease is low in premenopausal women, but it increases steadily in postmenopausal women, which suggests that estrogen may play an important role in the pathogenesis of hypertension and other cardiovascular diseases [39]. One of the key mechanisms underlying estrogen-mediated cardiovascular protection is the modulation of renin-angiotensin system (RAS). Indeed, previous studies have demonstrated that estrogen induces a down-regulation of AT_1_R gene expression in vasculatures, resulting in decreased vasoconstriction and blood pressure [40], [41]. Our current study shows an ER binding site at AT_1a_R promoter region, suggesting that estrogen can directly regulate AT_1_R gene promoter activity in vasculatures. Furthermore, our present finding that a lower methylation at the estrogen receptor binding site within the AT_1a_R promoter in response to nicotine exposure, suggests that nicotine-induced demethylation may counteract the inhibitory effect of estrogen on AT_1_R promoter activity, resulting in increased AT_1_R gene expression.

Numerous studies have shown that the effect of fetal stress on DNA methylation is a species-, gender- tissue-, organelle-, and gene-dependent [22], [42]–[45]. In contrast to the hypomethylation at specific CpG islands in AT_1_aR promoter, nicotine treatment selectively increased the methylation levels at −444 and +11 CpG locus in AT_2_R promoter in vasculatures. This suggests that the effect of perinatal nicotine exposure on DNA methylation is gene-specific. In a similar animal model, previous studies have demonstrated that perinatal nicotine exposure decreases DNA methylation at AT_2_R promoter and AT_2_R protein repression of neonatal brain in male offspring but not in female offspring [23]. These studies suggest that the effect of perinatal nicotine exposure on DNA methylation is, at least gene- and gender-dependent. The molecular mechanisms of perinatal exposure to nicotine on DNA methylation in adulthood are unclear. Given the fact that perinatal exposure to nicotine enhances vascular oxidative stress in offspring [11], we speculate that the heightened oxidative stress may directly alter methylation pattern at ATR promoter region, resulting in change of its gene expression, which open a broad door for us to further study of the potential molecular mechanisms underlying nicotine-induced DNA methylation of ATR gene.

**Figure 7:**
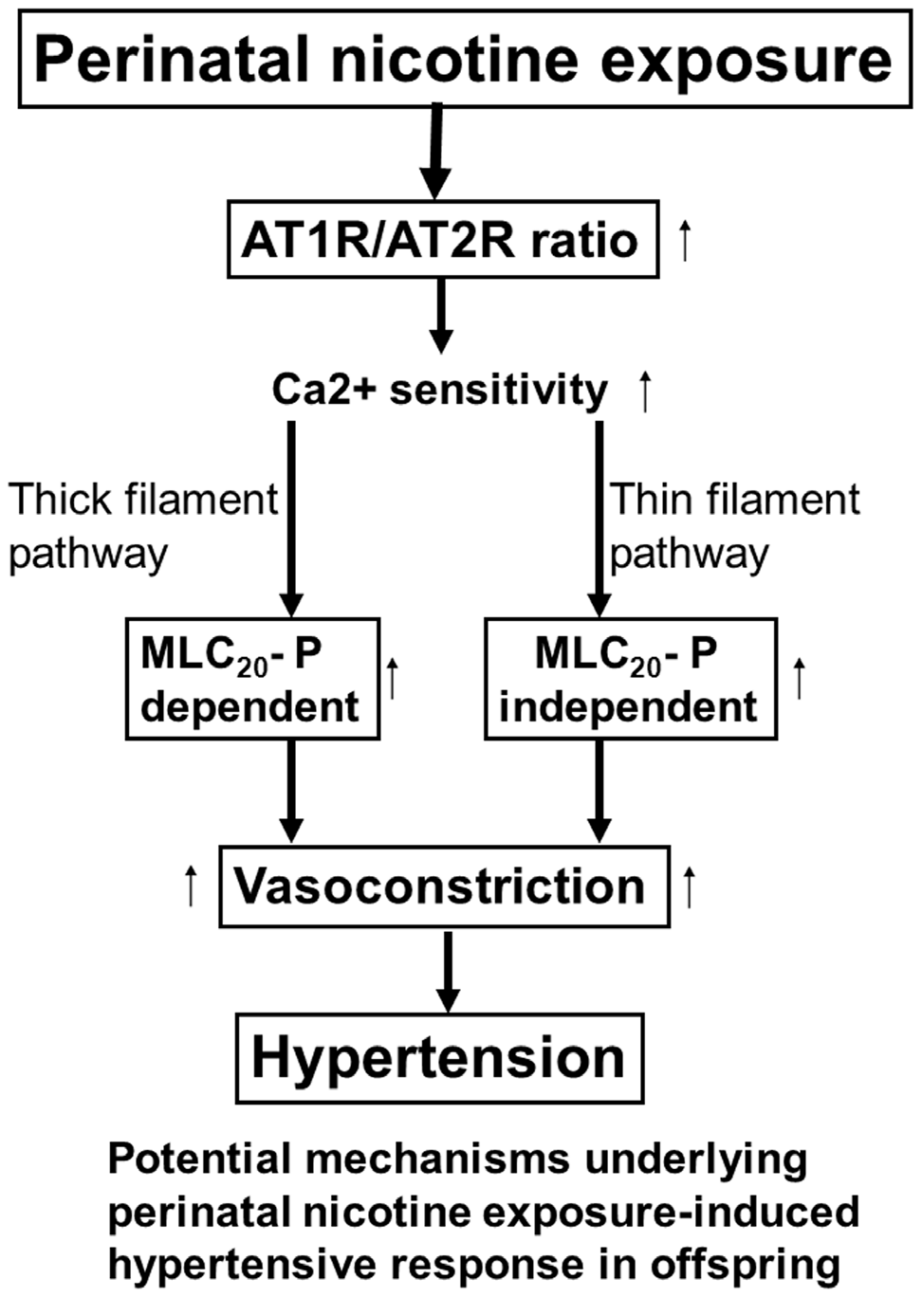
Diagram of potential mechanisms underlying fetal nicotine exposure-induced hypertensive response in adult offspring. Perinatal nicotine exposure enhances AT_1_R and attenuate AT_2_R gene expression through DNA methylation mechanism. Nicotine-mediated enhanced AT_1_R/AT_2_R ratio predominately heightens Ca^2+^ sensitivity of myofilaments, leading to exaggerated vasoconstriction through both MCL_20_ phosphorylation-dependent (thick filament) and –independent (thin filament) signaling pathways and development of hypertensive phenotype in adult offspring.

In conclusion, the present studies provide evidence of a novel mechanism of the selectively altered methylation of specific CpG at AT_1_R/AT_2_R gene promoter in epigenetic modification of gene expression patterns in the developing vasculature and the resultant increase in vascular contractility in adult offspring caused by perinatal nicotine exposure. As shown in Figure 7 (diagram), perinatal nicotine exposure selectively enhanced AT_1_R but decreased AT_2_R gene expression patterns through DNA methylation mechanism. The increased AT_1_R/AT_2_R ratio enhanced both thick-filament regulatory pathway (*i.e*. MLC_20_-P -dependent signaling) and thin-filament regulatory pathway (*i.e.* MLC_20_-P -independent signaling), resulting in heightened vascular contractility and development of hypertensive phenotype in adult offspring. Given the fact that maternal tobacco cigarette or e-cigarette smoking and use of nicotine gum and patch during pregnancy is a major stress to the developing fetus and newborn, which may cause an increased risk of hypertension and other cardiovascular disease in adulthood, further studies on the epigenetic regulation of AT_1_R and AT_2_R gene expression patterns in the developing cardio-vasculature should provide more insights into molecular mechanisms in maternal smoking-related cardiovascular disease, and may suggest new insights of therapeutic strategies in the treatment of cardiovascular dysfunction in adulthood.
